# Genetic diversity and connectivity in the East African giant mud crab *Scylla serrata*: Implications for fisheries management

**DOI:** 10.1371/journal.pone.0186817

**Published:** 2017-10-24

**Authors:** Cyrus Rumisha, Filip Huyghe, Diary Rapanoel, Nemo Mascaux, Marc Kochzius

**Affiliations:** 1 Sokoine University of Agriculture, Solomon Mahlangu College of Science and Education, Department of Biosciences, Morogoro, Tanzania; 2 Vrije Universiteit Brussel, Department of Biology, Marine Biology, Brussels, Belgium; National Cheng Kung University, TAIWAN

## Abstract

The giant mud crab *Scylla serrata* provides an important source of income and food to coastal communities in East Africa. However, increasing demand and exploitation due to the growing coastal population, export trade, and tourism industry are threatening the sustainability of the wild stock of this species. Because effective management requires a clear understanding of the connectivity among populations, this study was conducted to assess the genetic diversity and connectivity in the East African mangrove crab *S*. *serrata*. A section of 535 base pairs of the cytochrome oxidase subunit I (COI) gene and eight microsatellite loci were analysed from 230 tissue samples of giant mud crabs collected from Kenya, Tanzania, Mozambique, Madagascar, and South Africa. Microsatellite genetic diversity (H_e_) ranged between 0.56 and 0.6. The COI sequences showed 57 different haplotypes associated with low nucleotide diversity (current nucleotide diversity = 0.29%). In addition, the current nucleotide diversity was lower than the historical nucleotide diversity, indicating overexploitation or historical bottlenecks in the recent history of the studied population. Considering that the coastal population is growing rapidly, East African countries should promote sustainable fishing practices and sustainable use of mangrove resources to protect mud crabs and other marine fauna from the increasing pressure of exploitation. While microsatellite loci did not show significant genetic differentiation (p > 0.05), COI sequences revealed significant genetic divergence between sites on the East coast of Madagascar (ECM) and sites on the West coast of Madagascar, mainland East Africa, as well as the Seychelles. Since East African countries agreed to achieve the Convention on Biological Diversity (CBD) target to protect over 10% of their marine areas by 2020, the observed pattern of connectivity and the measured genetic diversity can serve to provide useful information for designing networks of marine protected areas.

## Introduction

The giant mud crab (*Scylla serrata*) is widely distributed in the Indo-Pacific and it is the only *Scylla* species found at African shores [1]. The crabs provide an important source of income and food to coastal communities in East Africa [2]. Adult and juvenile mud crabs inhabit muddy estuaries and mangrove ecosystems where they can be found buried in mud or taking shelter in burrows at low tide [3,4]. Mated females migrate offshore to spawn because offshore waters provide optimum salinity for larval development and greater chances for dispersal [5]. After hatching, the planktonic larvae undergo a series of up to five moults for a period of two to three weeks [6]. During this period, the larvae are susceptible of being transported by currents and tides to coastal areas where they settle in sheltered areas among mangroves and seagrass. Therefore, currents and tides can influence larval availability. Stock structure and population persistence depends greatly on successful larval settlement and recruitment into the adult population [4]. Knowledge of the patterns of connectivity between sites is crucial for the identification of genetically meaningful management units.

Settlement and recruitment of marine organisms are complex processes, influenced by the interaction of multiple biotic and abiotic factors which operate at different temporal and spatial scales [7]. To identify suitable areas for settlement, larvae of most crustaceans, including *Scylla serrata*, rely on chemical cues produced by adults, predators, and certain macrophytes [8]. However, the ability of olfactory receptors to detect such cues can be seriously affected by the presence of contaminants in the environment [9]. Stock structure can also be affected by overexploitation and habitat alteration. Due to rapid population growth in coastal areas in East Africa, exploitation of mud crabs and other sea food has increased drastically. The rapidly expanding tourism industry and export trade has also led to increased demand and exploitation of mud crabs in the region [10]. As a result, the preferred market size has decreased consistently from more than 1 kg two decades ago to the current size of 0.5 kg [2]. A previous study reported that mud crab catches in the region are dominated by young crabs of 75 mm carapace length, suggesting that few juveniles are able to recruit into the spawning population [11]. The reduction of the spawning population can have serious effects on genetic diversity and sustainability of the mud crab fishery. The collection of juvenile mud crabs for utilisation in aquaculture [12] also puts more pressure on the wild stock and it is likely to exacerbate overexploitation, because this kind of farming is expanding drastically.

The growing coastal population is also threatening the sustainability of mud crab fisheries due to increased incidences of pollution [13,14], and mangrove degradation [15]. In general, 1.25% of the existing African mangrove forest is lost each year [16]. Habitat loss and fragmentation can influence the genetic structure of populations by limiting dispersal capabilities of species [17], which leads to reduced fitness of the population and cases of localised extinction. Although giant mud crabs have very high dispersal capacities [4], they might, over time, suffer these consequences [18]. Such consequences were reported in other mangrove fauna in the region [19,20]. Since genetic diversity is the basis for adaptation, management of genetic diversity is crucial for maintaining the sustainability of marine resources. However, conservation and management of genetic diversity require a clear understanding of the pattern of connectivity among populations.

In 2010, East African countries agreed to implement the UN Convention on Biological Diversity (CBD) strategic plan for biodiversity 2011–2020, which targets to protect over 10% of marine areas by 2020 [21]. Efforts have been taken because up to now, 8.7% of the continental shelf in Kenya, 8.1% in Tanzania, and 4.0% in Mozambique have been designated as marine protected areas (MPAs) [22]. The MPAs provide spatial escape for intensely exploited species, act as buffers against management miscalculations and unforeseen or unusual conditions, and they are expected to act as centres for dispersion of propagules to surrounding areas [23]. Assessment of the effectiveness of the existing MPAs and establishment of new MPAs require a clear understanding of the patterns of connectivity in the study area. Recent studies did not detected significant genetic differentiation among populations of fiddler crabs *Uca annulipes* [24] and littorinid gastropods (*Littoraria scabra* and *Littoraria glabrata*) [25] along the East African coast. The significant genetic differentiation among the East African *S*. *serrata* populations documented in a previous study [26] was not confirmed in a more recent study which used a larger number of individuals [27]. Both studies used a fragment (535 base pairs) of the mitochondrial cytochrome oxidase subunit I gene (COI) to analyse genetic variability and connectivity. In order to obtain a better resolution of the genetic population structure of *S*. *serrata* in the WIO, the present study was conducted using both mitochondrial and microsatellite DNA markers.

## Materials and methods

### Study area

**Fig 1:**
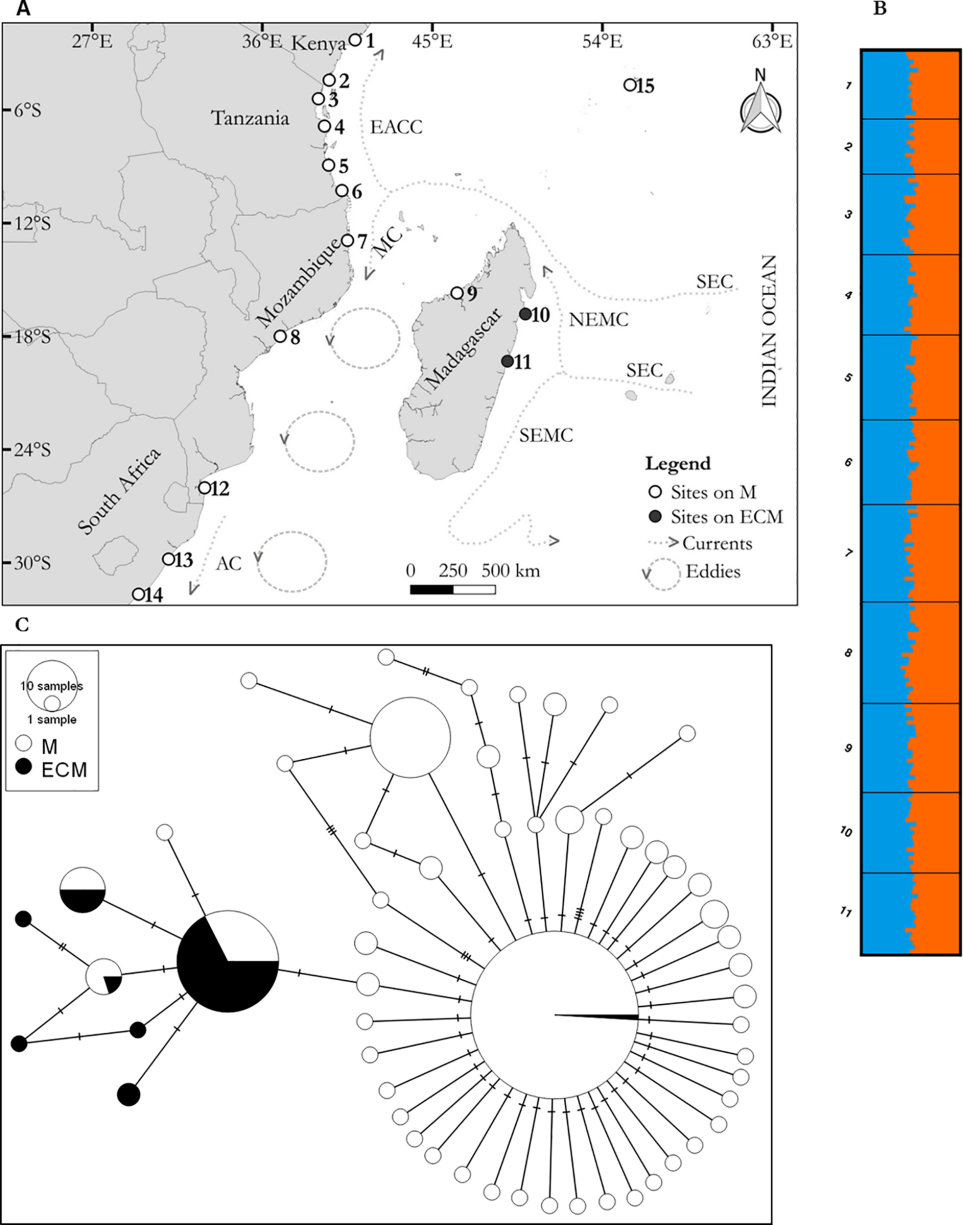
**A**. Map of the East African coast showing sample sites. SEC = South Equatorial Current, EACC = East African Coast Current, MC = Mozambique Current, NEMC = Northeast Madagascar Current, SEMC = Southeast Madagascar Current, AC = Agulhas Current. Main ocean currents were drawn according to [31]. **B**. Bar charts showing the likelihood of individual genotypes of belonging to different groups inferred by STRUCTURE analysis. **C**. Haplotype network of partial cytochrome oxidase subunit I sequences. Each circle represents a haplotype. Size of each circle is proportional to the number of individuals carrying each haplotype. The central haplotype represents 109 sequences. Hatch marks = mutations. EA = sites on mainland East Africa, West coast of Madagascar, and Seychelles. ECM = East Coast of Madagascar.

The study was conducted in East African coastal waters. This coastal zone is characterised by mangrove forests, fringing coral reefs, sand beaches, and rock outcrops [28]. Molluscs, mudskippers, sesarmid crabs, fiddler crabs, and giant mud crabs are commonly found in the mangrove forests [29,30]. The climate of the region has two alternating and distinctive seasons, influenced by the southern and the northern monsoons, which have a marked effect on winds, rainfall, as well as air and water temperature [1]. Ocean currents are driven by trade winds, which are greatly influenced by the movement of the thermal equator (intertropical convergence zone). The currents have a direct influence on nutrient transport and potentially on larval dispersal. The westward South Equatorial Current (SEC) splits at around 17°S in front of the East coast of Madagascar and flows northward as the Northeast Madagascar Current (NEMC) and southward as the Southeast Madagascar Current (SEMC) [31]. The extension of the SEC northwest of Madagascar reaches the African coast around 11°S, where it splits into the northward East African Coast Current (EACC) and the southward Mozambique Current (MC) [32]. Flow in the Mozambique channel is dominated by eddies which propagate southward into the Agulhas Current (AC) (Fig 1).

### Sampling

Sampling of giant mud crabs (*S*. *serrata*) was conducted between 2011 and 2015. Tissue samples of 230 individual giant mud crabs were collected from mangrove forests in Kenya, Tanzania, Mozambique, Madagascar, and South Africa (Fig 1 and Table 1). The mud crabs were collected at low tide with the help of local fishermen or bought at landing sites. A section of the pereopod tissue was collected from each animal and preserved in 99.9% ethanol for further analysis.

**Table 1:** Number of giant mud crabs (*Scylla serrata*) collected from mangrove forests at the Western Indian Ocean. COI = Cytochrome oxidase subunit I sequences analysed, COI previous study = COI sequences taken from previous studies [27,33].

Site	Site name	Coordinates		Samples		
		Longitudes (° E)	Latitudes (° S)	Microsatellite Samples	COI this study	COI previous studies
1	Lamu, Kenya	40.91	2.29	16	14	30
2	Gazi, Kenya	39.54	4.42	13	14	30
3	Pangani, Tanzania	38.97	5.41	32	31	-
4	Dar es Salaam, Tanzania	39.29	6.86	20	20	-
5	Kilwa, Tanzania	39.51	8.93	20	20	-
6	Mtwara, Tanzania	40.21	10.27	20	20	-
7	Pemba, Mozambique	40.51	12.92	23	22	-
8	Quelimane, Mozambique	36.95	18.00	24	25	-
9	Mahajanga, Madagascar	46.31	15.70	21	21	-
10	St Marie, Madagascar	49.93	16.82	19	18	-
11	Vatomandry, Madagascar	48.98	19.32	19	20	-
12	Inhaca, Mozambique	32.95	26.03	-	-	28
13	Durban, South Africa	31.04	29.81	-	-	11
14	Kwa Zulu Natal, South Africa	29.45	31.67	-	5	
15	Mahe island, Seychelles	55.47	4.67	-	-	26
	** Total**			**227**	**230**	**125**

### Ethics statement

Permission to collect samples was provided by the Tanzania Commission for Science and Technology (COSTEC), Tanzania Ministry of Agriculture, Livestock and Fisheries, the University of Tuléar (Madagascar), and the School of Marine and Coastal Sciences, Eduardo Mondlane University.

### Laboratory analyses

#### DNA extraction

Total DNA was extracted from the collected tissues (20–30 mg) by using the E.Z.N.A. Tissue DNA Kit (Omega Bio-Tek Inc., Norcross, USA). Tissue lysis, DNA extraction, and purification were performed according to the manufacturer’s protocol. Agarose gel electrophoresis was performed to check the quality of the DNA extracts. Agarose gel electrophoresis was performed using the procedures outlined in a previous study [20].

#### Polymerase chain reactions

Polymerase chain reactions (PCR) were performed using an MJ research PTC 200 Peltier thermocycler. Multiplex PCR was performed to assess microsatellite polymorphism. Two multiplex systems containing eight microsatellite markers were developed using previously published markers (Table 2)[34–36]. The software Multiplex Manager ver. 1.2 [37] was used to organise the primers into two multiplex sets. Multiplex PCR reactions were performed using the Type-it Microsatellite PCR Kit (QIAGEN Inc., Valencia, CA, USA). Optimisation and thermocycling profiles were conducted according to the manufacturer’s protocol. Agarose gel electrophoresis was performed to assess yield and quality of the PCR products. Fragment analysis was performed by using an Applied Biosystems 3730 capillary sequencer. GeneScan 500 LIZ was used as a size standard. The obtained microsatellite fragments were genotyped with the software GeneMarker ver. 2.2.0 (SoftGenetics LLC, Oakwood, USA). The program CONVERT ver. 1.3.1 [38] was used to reformat the obtained genotypic data in order to generate input files for population genetic software packages used in subsequent analyses. PCR and fragment analysis were repeated for samples that produced unclear genotypes.

A fragment (557 bp) of the COI gene was also amplified using the primers mtd10 5´ TTGATTTTTTGGTCATCCAGAAGT 3´ [39] and C/N 2769 5´ TTAAGTCCTAGAAATGTTRGGGA 3´ [33]. The PCR were done in a total volume of 25 μL containing 10 ng of the DNA template, 0.25 U of the *Thermus aquaticus* DNA polymerase, 0.2 μM of each primer, 0.2 mM DNTP, 3 mM MgCl_2_, 1x Taq buffer, and 0.4 mg bovine serum albumin. The PCR profiles included an initial denaturation step of 5 min at 95°C, followed by 35 cycles of 30 s at 95°C, 30 s at 50°C, and 1 min at 72°C. A final extension step of 10 min at 72°C was added to ensure complete amplification. Agarose gel electrophoresis was performed to assess the quality of the PCR products. Sequencing of both strands of the fragment of COI gene was done with an ABI 3700 XL sequencer. For each sample, the obtained forward and reverse sequences were edited and aligned using the ClustalW algorithm as implemented in MEGA ver. 6.0 [40] to generate consensus sequences (557 base pairs). The same software was used to translate the nucleotide sequences into amino acid sequences using the invertebrate mitochondrial genetic code. This was done in order to ensure that functional mitochondrial DNA was obtained and not a nuclear pseudogene [41].

**Table 2:** Primers used to amplify microsatellite loci in the giant mud crab *Scylla serrata* from the Western Indian Ocean. Cy3 = Cyanine3. Dye = fluorescent dye, Na = number of alleles, Ta = annealing temperature.

	Locus	Repeat motif	Primer sequence (5ꞌ-3ꞌ)	Size (bp)	Na	Dye
Multiplex 1 (Ta = 50°C)	Scpa-INI-SSR	(AG)_31_	F: CTGTCTGTCCCTCGCGTCC	167–215	22	HEX
			R: TTCTCTCCCTTTTGAGCGAATAAG			
	Scse53-1	(CA)_32_	F: CCGTCACTTCACAGTATA	236–240	2	Cy3
			R: GTTTTCATTTGAGTTTCC			
	Scse43-1	(TG)_15_	F: GAAATCTGAGCTGCCAATC	222–240	10	ROX
			R: CACCCATCCAAGTACCAA			
Multiplex 2 (Ta = 54.2°C)	Scse96-1	(GAAGG)_10_	F: CTTCCTCACCGTCCCTAT	270–285	4	6FAM
			R: CTCTGTTGCCTAATTCCTC			
	Scpa-CB-SSR	(TG)_17_	F: CAGTGCAAGGCAAGTCAGGATAC	264–296	15	ROX
			R: AGTTCTGGAAGCATGCAATACTGAC			
	SCY38	(CA)14	F: CAGACACTCAAGTCTCACCTGC	233–245	7	HEX
			R: CAGAATGGTTAATGGGGGG			
	SCY12	(CA)16	F: AGACCTCTCTCCCTTCCTGC	201–211	6	Cy3
			R: GGTGAACCTGCTTGGCAC			
	SCY23	(CA)11	F: TGACAGTTGGTAGAGGCGC	113–117	3	Cy3
			F: GTCTAGCTGAGAGGGCGATG			

### Data analyses

#### Analysis of genetic diversity

Microsatellite based estimates of the observed and expected heterozygosity were determined with the software GenAlEx ver. 6.5 [42]. The same software was used to test for departure from the Hardy-Weinberg equilibrium (HWE). Allelic richness was estimated with the program FSTAT ver. 2.9.3 [43]. The same program was used to estimate F_IS_ (within sub-population inbreeding coefficient) and to test whether it is significantly different from zero. Prior to these analyses, samples with missing data at three or more loci were removed from the data set. The data set was also checked for null alleles, large allele drop out, and scoring errors due stuttering using the software Micro-Checker ver. 2.2 [44].

A total of 230 COI sequences were obtained from the analysed tissues. Alignment of the edited sequences was performed with MEGA ver. 6.1 [40] to generate a multiple alignment with 535 base pairs. Estimates of genetic diversity, such as the number of haplotypes, haplotype diversity, current nucleotide diversity (θ_π_, based on pairwise differences), and historical nucleotide diversity (θ_w_, based on number of segregating sites) were calculated with the program DnaSP ver. 5.10 [45].

#### Population structure and demographic history

The analysis of molecular variance (AMOVA) of the microsatellite data was performed with the software Arlequin ver. 3.5.1.2 [46], in order to determine the pattern of differentiation between sample sites. Since the markers displayed multiple alleles, correlation analysis between the single locus G_ST_ values and the within subpopulation genetic diversity (Hs) was performed using the computer program CoDiDi (Correlation between Diversity and Differentiation) ver. 1.0 [47]. This was done in order to determine if G_ST_ is an appropriate measure of genetic differentiation for the sampled populations. Generally, G_ST_ gives correct estimates of genetic differentiation if the effect of mutation is lower than other demographic forces. Mutation effects are lower than other demographic factors when the correlation between G_ST_ and Hs (within subpopulation expected heterozygosity) is not significant [47]. Pairwise comparison of G_ST_ was performed with GenAlEx ver. 6.5 [42], in order to determine the pattern of genetic differentiation between populations. The significance of G_ST_ values was determined according to the Holm-Bonferroni sequential procedure [48]. To test whether individuals clustered according to geographical origin, a Bayesian analysis implemented in the software STRUCTURE ver. 2.3.4 [49] was performed, testing for different numbers of clusters (*k*) in the dataset and giving the corresponding probabilities. STRUCTURE HARVESTER ver. 0.6.94 was used to infer the optimal *k* through the Δ *k* statistic, which is based on the rate of change of log probability of the data between successive *k*–values [50].

The 230 analysed COI sequences were combined with 125 previously published sequences [27,33], to form a combined data set with 355 sequences (Table 1). The software MEGA ver. 6.0 [40] was used to align the sequences. The program FaBox DNA collapser ver. 1.41 [51] was used to collapse the aligned sequences into haplotypes and to create input files for subsequent analyses. Analysis of Molecular Variance (AMOVA) of the sequences was performed in order to analyse the partitioning of the total genetic variation and to estimate the fixation index. This was done by using the software Arlequin ver. 3.5.1.2 [46]. The same software was used to compare populations by computing pairwise F_ST_ values, which were calculated from haplotype frequencies. The significance of pairwise F_ST_ values was calculated by 10,000 random permutations of haplotypes between populations. The F_ST_ p-values were adjusted using the Holm-Bonferroni sequential procedure [48]. Hierarchical AMOVA was performed to determine if there is a significant genetic break between groups of populations. The significance of the population fixation indices (F_ST_ and Φ_ST_) was determined with 10,000 permutations. A minimum spanning haplotype network was constructed with the software PopART ver. 1.7 [52] to examine the relationship between haplotypes. The mutation-scaled effective population size Θ (2Neμ) and the mutation-scaled migration rates (M = m/μ) (where Ne = effective population size, m = immigration rate per generation, μ = mutation rate per generation) were estimated using the program MIGRATE-N ver. 3.6.11 [53]. The program was run based on a full migration matrix model and Bayesian inference. The parameters Θ and M were estimated based on an exponential posterior distribution and a single long chain run consisting of 50 000 recorded steps, burn-in of 100 000, and four heated chains (static heating scheme) with temperatures 1.00, 1.50, 3.00 and 1 000 000. Prior to this, three replicate runs (without heating) were performed to estimate the boundaries of Θ and M. The number of immigrants per generation (2Nem) was obtained by multiplying Θ and M [53].

Fu’s Fs [54] and Tajima’s D [55] tests of neutrality were performed to evaluate the demographic history of the studied populations. Mismatch distribution analysis was performed to estimate the parameters of the sudden expansion model such as the sum of the squared deviation, the Harpending's Raggedness index, and the time since expansion [56].

## Results

### Genetic diversity

The eight analysed microsatellite loci did not show significant evidence of large allele drop out or scoring errors due to stuttering. In addition, the analysed loci did not show significant evidence of null alleles in all sampled populations, except locus Scse43-1 at site 4. The loci Ssse96-1 and Scpa-INI-SSR showed significant deviation from the HWE at site 3. The locus Scse43-1 showed significant departure from the HWE at sites 4 and 7. Significant departure from the HWE was also shown by the loci SCY38 and SCY12 at sites 6 and 10, respectively. The locus Scse53-1 was monomorphic at all sites, except site 1 (Table 2). The total number of alleles ranged between 2 and 22. Expected heterozygosity ranged between 0.561 and 0.601 (Table 3). The within sub-population inbreeding coefficients (F_IS_) were not significantly different from zero (p > 0.00057 (adjusted nominal level)).

**Table 3:** Indices of microsatellite genetic diversity in the East African giant mud crab *Scylla serrata*. N = sample size, Ar = allelic richness, H_o_ = observed heterozygosity, H_e_ = expected heterozygosity, F_IS_ = within sub population inbreeding coefficient. Cy3 = Cyanine3. For sites see Table 1 and Fig 1.

Site	N	Ar	H_o_	H_e_	F_IS_
1	16	4.5	0.57	0.591	0.04
2	13	4.5	0.61	0.587	-0.04
3	32	4.9	0.57	0.596	0.04
4	20	4.5	0.61	0.597	-0.02
5	20	4.2	0.64	0.561	-0.14
6	20	4.5	0.53	0.583	0.10
7	22	4.8	0.59	0.593	0.00
8	24	4.9	0.59	0.601	0.01
9	19	4.3	0.58	0.583	0.00
10	18	3.9	0.54	0.576	0.07
11	21	3.7	0.58	0.567	-0.01

A total of 230 COI sequences each with 535 base pairs were obtained. Diversity indices were calculated only for sites with at least 14 sequences. The analysed sequences showed 40 haplotypes. The highest haplotype diversity was observed at sites 2 and 8 (Table 4). The lowest haplotype diversity was measured in samples from site 11. The current nucleotide diversity was generally low as it ranged between 0.07% (site 11) and 0.32% (site 8). In addition, the current nucleotide diversity was generally low than the historical nucleotide diversity (θ_π_ < θ_w_).

**Table 4:** Indices of molecular diversity in the East African giant mud crab *Scylla serrata* based on mitochondrial cytochrome oxidase subunit I sequences. N = sample size, n_h_ = number of haplotypes, h = haplotype diversity, θ_π_ = current nucleotide diversity, θ_w_ = historical nucleotide diversity. For sample sites, see Fig 1 and Table 1.

Sites	1	2	3	4	5	6	7	8	9	10	11	Total
N	14	14	31	20	20	20	22	25	21	18	20	**225**
n_h_	5	7	10	4	8	5	5	11	9	8	3	**40**
h	0.59	0.85	0.66	0.28	0.59	0.66	0.62	0.85	0.65	0.64	0.35	**0.75**
θ_π_ (%)	0.26	0.29	0.22	0.13	0.18	0.24	0.18	0.32	0.19	0.22	0.07	**0.29**
θ_w_ (%)	0.41	0.29	0.52	0.32	0.42	0.37	0.31	0.50	0.53	0.44	0.11	**1.15**

### Demographic history

**Fig 2:**
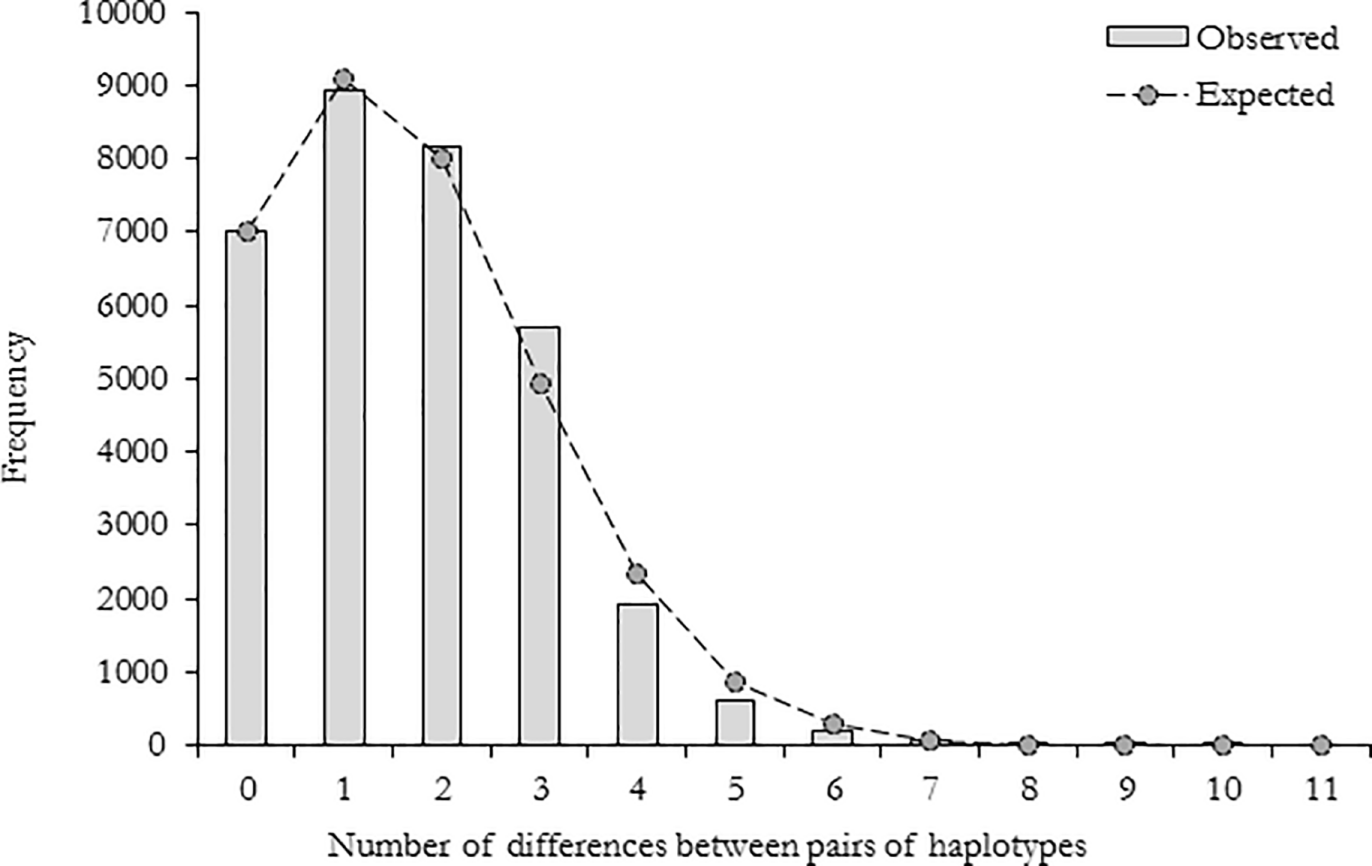
Pairwise mismatch distribution showing a unimodal distribution of the cytochrome oxidase subunit I haplotypes in the East African giant mud crab *Scylla serrata*.

A multiple alignment of the 230 sequences obtained during this study and the 125 previously published COI sequences was performed. The sequences were collapsed with the program FaBox DNA collapser ver. 1.41 [51] to generate 57 haplotypes (Table 5). The haplotype sequences were submitted to GenBank (accession numbers for haplotypes 1–57 = MF496045—MF496101). Fu’s Fs and Tajima’s D test of the pooled samples showed significant deviation from the neutral evolution hypothesis (Tajima’s D = -2.36, p < 0.001: Fu’s Fs = -27.48, p < 0.001). Mismatch distribution of the pooled samples produced a unimodal distribution, supporting the null hypothesis of population expansion (Fig 2). The raggedness index and sum of squared deviations (SSD) showed that the null hypothesis of population expansion cannot be rejected (raggedness index = 0.025, p > 0.05: SSD = 0.00087; p > 0.05). Fu’s Fs and Tajima’s D test were also performed for each population and they indicated significant deviation from the hypothesis of neutral evolution for all sampled populations, except populations at sites 6, 7, 11, 13, 14, and 15 (Table 6). The raggedness index for each population was not significant except for the population at site 8.

**Table 5:** Distribution of the cytochrome oxidase subunit I haplotypes in the East African giant mud crab *Scylla serrata*. The number below each haplotype is proportional to the number of individuals carrying each haplotype, n_h_ = number of haplotypes (GenBank accession numbers for haplotypes 1–57 = MF496045—MF496101).

Site	n_h_	Distribution of haplotypes																
1	12	h1	h10	h11	h12	h13	h16	h17	h18	h19	h26	h27	h28					
		30	4	1	1	1	1	1	1	1	1	1	1					
2	17	h1	h2	h3	h4	h5	h6	h7	h8	h9	h10	h14	h15	h27	h29	h30	h31	h32
		20	1	1	1	1	1	1	1	2	5	1	2	3	1	1	1	1
3	9	h1	h5	h10	h33	h34	h35	h36	h41	h43								
		18	1	4	1	1	1	1	2	1								
4	4	h1	h36	h37	h38													
		17	1	1	1													
5	8	h1	h10	h30	34	h39	h40	h41	h42									
		13	1	1	1	1	1	1	1									
6	5	h1	h10	h13	h19	h27												
		11	3	1	1	4												
7	5	h1	h11	h15	h37	h44												
		12	1	1	7	1												
8	11	h1	h15	h27	h30	h31	h40	h45	h46	h47	h48	h49						
		4	1	9	2	1	1	1	2	1	1	2						
9	9	h1	h15	h27	h33	h45	h54	h55	h56	h57								
		13	1	1	1	1	1	1	1	1								
10	8	h1	h10	h15	h30	h50	h51	h52	h53									
		1	11	1	1	1	1	1	1									
11	3	h10	h15	h52														
		16	3	1														
12	8	h1	h10	h15	h19	h20	h21	h22	h23									
		18	3	1	1	1	1	2	1									
13	1	h1																
		11																
14	2	h1	h5															
		4	1															
15	5	h1	h7	h10	h24	h25												
		18	1	5	1	1												

**Table 6:** Parameters estimated under the selective neutrality tests and the sudden expansion model for the East African giant mud crab (*Scylla serrata*) based on cytochrome oxidase subunit I sequences. D = Tajima's D, FS = Fu's FS, HRI = Harpending's raggedness index, SSD = sum of squared deviations, p = p-values. For sample sites, see Table 1 and Fig 1.

Sites	1	2	3	4	5	6	7	8	9	10	11	12	13	14	15
D	**-2.4**	**-1.9**	**-1.8**	**-1.9**	**-1.9**	-1.2	-1.3	-1.1	**-2.3**	**-1.7**	-0.8	**-1.7**	0.0	-0.8	-0.9
D p	0.00	0.01	0.01	0.01	0.01	0.12	0.08	0.13	0.00	0.02	0.25	0.02	1.00	0.29	0.20
FS	**-7.2**	**-13.7**	**-5.7**	-0.8	**-5.1**	-0.5	-1.2	**-5.8**	**-6.8**	**-4.6**	-0.8	**-3.8**	0.0	0.1	-1.1
FS p	0.00	0.00	0.00	0.24	0.00	0.33	0.14	0.00	0.00	0.00	0.20	0.00	N.A.	0.30	0.15
SSD	**0.39**	0.00	0.00	0.01	0.00	0.01	0.01	0.03	0.01	0.01	0.00	0.00	0.00	0.01	**0.36**
SSD p	0.00	0.60	0.85	0.36	0.80	0.57	0.21	0.07	0.32	0.58	0.47	0.86	0.00	0.79	0.00
HRI	0.2	0.0	0.0	0.3	0.1	0.1	0.1	**0.2**	0.1	0.0	0.2	0.0	0.0	0.2	0.2
HRI p	0.98	0.63	0.96	0.56	0.86	0.82	0.26	0.02	0.35	0.99	0.40	0.90	0.00	0.94	1.00

### Connectivity among populations

Correlation analysis showed that the association between the microsatellite based genetic differentiation (G_ST_) and the within subpopulation expected heterozygosity (Hs) is not significant (G_ST_ = 0.0093Hs—0.0023: r = 0.327, p > 0.05). The analysis of molecular variance of the microsatellite data showed that the variation among sites was not significant (F_ST_ = 0.00424, p > 0.05, 100172 permutations). STRUCTURE analysis did not detect meaningful genetic clusters (Fig 1). Apart from that, the analysis of molecular variance (AMOVA) of the COI sequences revealed significant genetic differentiation among sites (F_ST_ = 0.158, p < 0.001; Φ_ST_ = 0.238, p < 0.001). Pairwise comparison of F_ST_-values showed variable connectivity among the sample sites. With the exception of site 8, populations from Seychelles, Kenya, Tanzania, Mozambique, South Africa, and the West Coast of Madagascar did not show significant genetic differentiation (Table 7). Apart from that, a significant genetic break was observed for populations on the East coast of Madagascar (ECM) (sites 10 and 11), which were significantly differentiated from the other sample sites. This was confirmed by hierarchical AMOVA, which showed significant genetic differentiation between populations on the ECM and other sampled populations (F_CT_ = 0.361, p < 0.01: Φ_CT_ = 0.564, p < 0.01). The observed pattern of genetic differentiation was also revealed in the haplotype network (Fig 1). The network showed a star-like shape, with two main clusters of haplotypes joined to the main haplotype by few mutations. Haplotypes of populations on the ECM formed a separate cluster, which contained very few shared haplotypes (4 shared haplotypes and 4 private haplotypes). Sites from Kenya, Tanzania, and the Mozambique Channel showed high effective population size compared to other sites (Table 8). Estimates of the immigration rate showed high rate of immigration to Kenyan and Tanzanian mangroves and the lowest immigration rate to mangroves at the ECM.

**Table 7:** Pairwise F_ST_-values derived from pairwise comparison of cytochrome oxidase subunit I sequences of giant mud crabs (*Scylla serrata*) in the Western Indian Ocean. Bold values are significant after Holm-Bonferroni sequential correction. For sample sites, see Table 1 and Fig 1.

Sites	1	2	3	4	5	6	7	8	9	10	11	12	13	14
2	0.03													
3	0	0												
4	0.02	0.10	0.06											
5	-0.02	0.01	-0.02	0.02										
6	0.01	-0.01	0.00	0.11	0.01									
7	0.07	0.03	0.06	0.15	0.06	-0.01								
8	**0.23**	0.09	**0.17**	**0.33**	**0.19**	0.10	0.07							
9	-0.01	0.01	-0.01	0.04	-0.02	0.01	0.03	**0.16**						
10	**0.36**	**0.20**	**0.27**	**0.52**	**0.34**	**0.26**	**0.35**	**0.24**	**0.34**					
11	**0.50**	**0.34**	**0.42**	**0.68**	**0.51**	**0.42**	**0.50**	**0.38**	**0.50**	0.01				
12	-0.01	0.01	-0.01	0.04	-0.02	0.01	0.07	**0.20**	-0.01	**0.32**	**0.47**			
13	0.07	0.17	0.13	0.01	0.10	0.19	0.23	**0.40**	0.11	**0.60**	**0.78**	0.10		
14	-0.05	0.01	-0.03	-0.04	-0.06	0.01	0.05	0.21	-0.05	0.41	**0.63**	-0.04	0.17	
15	-0.01	0.03	-0.01	0.04	-0.01	0.02	0.10	**0.24**	0.02	**0.33**	**0.49**	-0.01	0.11	-0.02

**Table 8:** Mutation-scaled effective population size (Θ) and the mutation-scaled immigration rates (M = m/μ) in the giant mud crabs *(Scylla serrata*) from the Western Indian Ocean. Migrants = number of immigrants (Θ times M). Group A = sites in Kenya and Tanzania, B = sites in the Mozambique channel, C = sites on the ECM, D = site 12–14, E = Seychelles.

Groups	Θ	Direction	M	Migrants	Total immigrants	Total emigrants
A	0.0445	B → A	300.5	4	7	10
B	0.0125	C → A	156.7	0	4	6
C	0.0028	D → A	432.4	3	3	2
D	0.0058	E → A	384.8	1	4	4
E	0.0017	A → B	66.1	3	4	1
		C → B	109.9	0		
		D → B	144.9	1		
		E → B	129.7	0		
		A → C	52	2		
		B → C	42.7	1		
		D → C	61.6	0		
		E → C	68.9	0		
		A → D	63.5	3		
		B → D	54.2	1		
		C → D	198.2	1		
		E → D	151.6	0		
		A → E	53.8	2		
		B → E	60.1	1		
		C → E	119.2	0		
		D → E	70.4	0		

## Discussion

### Genetic diversity

The East African *S*. *serrata* populations showed high mitochondrial DNA haplotype diversity and low nucleotide diversity (Table 4). This is due to the fact that most haplotypes differed from each other by very few mutations (one to five mutations, S1 Table). Similar observations were previously reported in *S*. *serrata* in the WIO [26,27] and the Western Pacific [33]. Similar findings were also reported in other mangrove fauna in the WIO [19,24]. The high haplotype diversity and low nucleotide diversity might indicate genetic bottleneck events, where most haplotypes became extinct, followed by population expansion [57]. The measured haplotype diversity (h = 0.75) and nucleotide diversity (θ_π_ = 0.29%) are low compared to the reported levels of genetic diversity in the mangrove crabs *Uca hesperiae* (h = 0.80 ± 0.02, θ_π_ = 0.25 ± 0.16%), *Perisesarma guttatum* (h = 0.85 ± 0.02, θ_π_ = 0.42 ± 0.25%), and *Neosarmatium africanum* (h = 0.82 ± 0.02, θ_π_ = 0.46 ± 0.26%) [58] from the WIO. Nevertheless, the measured indices of genetic diversity are higher than the reported levels of genetic diversity in the mangrove crab *Uca occidentalis* (h = 0–0.679, θ_π_ = 0–0.13% [24]: h = 0.19 ± 0.03, θ_π_ = 0.03 ± 0.04% [58]) from the WIO, but comparable to the reported levels of genetic diversity in *S*. *serrata* in the WIO [26,27,33,58]. The measured indices of microsatellite diversity are also comparable to reported levels of microsatellite diversity in *Scylla paramamosain* from the East China Sea [36]. Since genetic diversity is the raw material for evolution [59], these findings suggest that the studied population is genetically robust. However, the fact that current genetic diversity was low compared to historical genetic diversity indicates that the studied population experienced periods of overexploitation or historical bottlenecks.

### Demographic history

The Fu’s Fs and Tajima’s D test of the pooled sample showed significant deviation from the neutral evolution hypothesis (Tajima’s D = -2.36, p < 0.001: Fu’s Fs = -27.48, p < 0.001). When the hypothesis of neutral evolution was tested for each population, significant departure from the hypothesis were observed at all sampled populations, except the populations at sites 6, 7, 11, 13, 14, and 15 (Table 7). This indicates selection or demographic expansion of the *S*. *serrata* populations in the study area. Mismatch distribution of the pooled sample produced a unimodal distribution, supporting the null hypothesis of population expansion (Fig 2). The raggedness index and sum of squared deviations (SSD) showed that the null hypothesis of population expansion cannot be rejected (raggedness index = 0.025, p > 0.05: SSD = 0.00087; p > 0.05). The constructed haplotype network also support the null hypothesis of recent population expansion. The network produced a star like structure, with the central haplotypes surrounded by several haplotypes that show little base pair differences (Fig 1). This suggests that most haplotypes originated recently and it is indicative of recent population expansion from a small number of founders [60]. The time of expansion was estimated from the expansion parameter tau (τ), using the equation t = τ/2 μ, where μ is the rate of mutation. Using the estimated τ of 1.10625 and the COI mutation rate of 1.15% per million years [33], the time at which population expansion began was estimated to be about 90 thousand years ago. This time corresponds with the last glacial period which spanned from 125 to 14.5 thousands of years ago. The observed population expansion was probably due to sea level oscillations during this time [61].

### Connectivity among populations

The correlation between microsatellite genetic differentiation (G_ST_) and the within subpopulation heterozygosity was not significant (p > 0.05). This shows that single loci G_ST_-values are marker independent and that G_ST_ is the best estimate of genetic differentiation in the study area [47]. Apart from that, AMOVA of the microsatellite data did not detect significant genetic differentiation among sites (F_ST_ = 0.00424, p > 0.05, 100172 permutations). In contrast, COI showed significant genetic differentiation among sites (F_ST_ = 0.158, p < 0.05; Φ_ST_ = 0.238, p < 0.05). The contrasting patterns of genetic differentiation between nuclear and mitochondrial DNA are reported in several other species [62,63] and they can be due to a complex array of conditions that include selection, fluctuations in populations size, variations in sex ratio, and introgressive hybridisation [62,64]. Introgressive hybridisation from related species, does not account for the observed patterns, since *S*. *serrata* is the only *Scylla* species occurring in the study area [1]. In addition, the observed discrepancy might not be due to selection, because the neutrality tests showed no evidence of selection in the mtDNA (Table 7). While nuclear DNA is less likely to be affected by bottlenecks and rapid population expansions, mtDNA is more susceptible to these evolutionary forces due to its smaller effective population size [65,66]. Evidence of sudden expansion of the East African *S*. *serrata* populations was revealed by the mismatch analysis (Fig 2). This suggests that the observed discordance might be due to the varying effects of genetic drift on mitochondria and nuclear DNA. In addition, if there is no variations in sex ratio, the index of genetic differentiation is expected to be four times higher in mtDNA than nuclear DNA [67]. The ratio of mtDNA to nuclear DNA differentiation in the present study was 37 (0.157/0.00424). This suggests that the observed discrepancy in population differentiation between mitochondrial and microsatellite DNA is probably due to variation in sex ratio. Generally, in areas without sex-biased fishery, males giant mud crabs can outnumber females by three folds [68]. This is in line with what was observed during fieldwork, since males showed high abundance. High abundance of male *S*. *serrata* in East African mangroves was also reported in a previous study [11]. Therefore, the observed discordance is probably due to variation in sex ratio.

The analysis of molecular variance did not detect significant genetic differentiation among sites in the Seychelles, Kenya, Tanzania, Mozambique, and South Africa. The observed lack of genetic differentiation between these sites is in line with the findings of previous studies [27,58]. A similar pattern of genetic differentiation in this region was also reported in other mangrove fauna [24,25,58]. In contrast to these studies, this study detected significant genetic differentiation between sites at the East coast of Madagascar (ECM) and sites in mainland East Africa and the Seychelles (F_CT_ = 0.361, p < 0.05: Φ_CT_ = 0.564, p < 0.05). The fact that previous studies did not include samples from the ECM [24,25,58] can explain why no genetic differentiation was detected in previous studies. The fact that significant genetic differentiation between Mauritius Island and mainland East Africa was not detected in two previous studies [27,33] can be attributed to consequences of low sample size. The studies used only five sequences, representing only one haplotype from Mauritius. The observed genetic break between populations on the ECM and other sample sites is probably due to the influence of ocean circulation on larval transport and dispersal. Oceanic circulations in the region are influenced by trade and monsoon winds [1]. Because *S*. *serrata* has a planktonic larval stage, the SEC is expected to transport and disperse larvae from the ECM to East Africa through the EACC, MC and AC. The observed genetic differentiation indicate that there is limited larval exchange between sites in the ECM and sites in mainland East African and the Seychelles. This is supported by the measured mutation scaled immigration rate, which showed lowest rate of immigration to mangroves at the ECM (Table 8). The observed pattern in genetic differentiation is also supported by the haplotype network, which showed haplotypes from the ECM in a separate cluster, containing very few shared haplotypes (Fig 1). The patterns of currents can also account for the lack of genetic differentiation among sites on the coastline of East Africa. Circulation in East African coastal waters are influenced by the northward EACC, as well as the MC and eddies in the Mozambique channel, which propagate southward into the south-bound AC [31]. These currents are probably responsible for the dispersal of larvae among adjacent populations and thus accounting for the observed connectivity. This argument is supported the haplotype network, which showed that mangrove forests in the Mozambique channel, Kenya, Tanzania, South Africa and the Seychelles share the most common haplotypes (Fig 1).

### Implications for fisheries management

The study showed that the current genetic diversity is low compared to historical genetic diversity (θ_π_ < θ_w_). This shows that the studied population experienced bottlenecks in its recent history. Considering that indications of mud crab overexploitation and mangrove degradation are reported in the study area [2,11], measures aimed at enhancing sustainable use of resources should be strengthened. The observed limited gene flow between ECM and other sites indicate that protected mangroves and MPAs in the west coast of Madagascar and mainland East Africa cannot help to protect the biodiversity of mangroves in the ECM. Since Madagascar is planning to triple the extent of its MPAs by 2020, the observed patterns provide useful information for establishment of MPA networks around the island. Since giant mud crabs in the WIO are heavily exploited for food and trade, the observed low population size in the ECM and the Seychelles suggest that these areas require immediate attention. The mangroves in Kenya and Tanzania showed a high effective population size, which is maintained by a high rate of immigration from other mangroves in mainland East Africa. Estimates of migration rate showed highest number of immigrants to mangroves in this region (Table 8), indicating that overexploitation and degradation of some ecosystems is likely to affect recruitment and stock structure of adjacent ecosystems. Therefore, management efforts should strive to maintain connectivity among mangroves in this region. Since female giant mud crabs migrate offshore to spawn, management efforts should focus on both intertidal and offshore ecosystems.

## Conclusion

East African countries agreed to implement the UN Convention on Biological Diversity (CBD) strategic plan for biodiversity 2011–2020, which is targeting to achieve effective protection of 10% of the global marine ecoregions by 2020 [21]. Progress have been made, because so far 8.7% of the continental shelf in Kenya, 8.1% in Tanzania, and 4.0% in Mozambique has been designated [22]. The observed pattern of connectivity and the measured genetic diversity can serve to provide useful information for designing MPA networks for protection of biodiversity in the study area. Since signs of overexploitation and historical bottlenecks were observed at each site, special attention should be given to areas which showed low genetic diversity. Considering that the coastal population is growing rapidly, East African countries should promote sustainable fishing practices and sustainable use of mangrove resources to protect giant mud crabs and other marine fauna from the increasing pressure of exploitation.

## Supporting information

S1 Table Variable sites among the East African *Scylla serrata* COI haplotypes. (DOCX)
