# Supplementary material for: Genetic diversity and connectivity in the East African giant mud crab Scylla serrata: Implications for fisheries management
Source: PLoS One. 2017 Oct 24;12(10):e0186817. doi: 10.1371/journal.pone.0186817 (PMC5655608; doi:10.1371/journal.pone.0186817)
Supplement: S1 Table — (DOCX) [file pone.0186817.s001.docx]

**S1 Table. Variable sites among the East African *Scylla serrata* COI haplotypes.**

| 11111122222222233333333334444444555555555555  11335622234801345667700112223474567788001111111223  68925031936927340696470306251477554387909070236789251  1 ACAACCTAACATTGGTAGAATGTTCCTTTTGGGTACTTATAATTTATGCTAGT  2 .......G.............................................  3 .....................A........A........C.............  4 ...............C.....................................  5 ................................A....................  6 ...........C.........................................  7 .................................C...................  8 ...............................C.....................  9 .G...................................................  10 ..................................G..........G.......  11 ..........G..........................................  12 .................A.....C.................CC..........  13 ...........C......G..................................  14 ......................................C..............  15 ...........................C......G..........G.......  16 .............A.......................................  17 ........................T.......A....................  18 ...........C.......G.................................  19 .....................A........A......................  20 ......................C..............................  21 ...................................T.................  22 ................................................T....  23 ................G....................................  24 .....................A...............................  25 ...............................................A.....  26 ............................C..............A.........  27 ...........................................A.........  28 ...........................G.........A...........G...  29 ............C........................................  30 ..................................G........A.G.......  31 .............................................G.......  32 ....................C................................  33 .........A...........................................  34 G....................................................  35 ......C...........................G..........G.......  36 ....T................................................  37 ..............................T......................  38 .....................A.C...G..A........C.............  39 ........G............................................  40 .....T...............................................  41 ...................................................A.  42 ........................................G............  43 ...........C.................C.......................  44 .....................................A.....A.........  45 ..............A......................................  46 ..............................................C......  47 G..........................................A.........  48 ..G..................................................  49 ..................................................G..  50 ..................................G........AAG......A  51 ..................................G..A.....A.G.......  52 ...T..............................G..........G.......  53 ..................................G..A.......G.......  54 ......C..............................................  55 ..........................C..........................  56 .........................T...........................  57 ....................................C................... |
| --- |
